# The CXCL5/CXCR2 axis is sufficient to promote breast cancer colonization during bone metastasis

**DOI:** 10.1038/s41467-019-12108-6

**Published:** 2019-09-27

**Authors:** Ricardo Romero-Moreno, Kimberly J. Curtis, Thomas R. Coughlin, Maria Cristina Miranda-Vergara, Shourik Dutta, Aishwarya Natarajan, Beth A. Facchine, Kristen M. Jackson, Lukas Nystrom, Jun Li, William Kaliney, Glen L. Niebur, Laurie E. Littlepage

**Affiliations:** 1 Harper Cancer Research Institute, South Bend, IN USA; 2 Department of Chemistry and Biochemistry, University of Notre Dame, Notre Dame, IN USA; 3 Tissue Mechanics Laboratory, Department of Aerospace and Mechanical Engineering, Bioengineering Graduate Program, University of Notre Dame, Notre Dame, IN USA; 4 Department of Applied and Computational Mathematics and Statistics, University of Notre Dame, Notre Dame, IN USA; 5 Department of Orthopaedic Surgery and Rehabilitation, Loyola University Medical Center, Stritch School of Medicine, Maywood, IL USA; 6 Present Address: Department of Orthopaedic Surgery, Cleveland Clinic, Cleveland, OH USA

**Keywords:** Breast cancer, Cancer models, Metastasis

## Abstract

Bone is one of the most common sites for metastasis across cancers. Cancer cells that travel through the vasculature and invade new tissues can remain in a non-proliferative dormant state for years before colonizing the metastatic site. Switching from dormancy to colonization is the rate-limiting step of bone metastasis. Here we develop an ex vivo co-culture method to grow cancer cells in mouse bones to assess cancer cell proliferation using healthy or cancer-primed bones. Profiling soluble factors from conditioned media identifies the chemokine CXCL5 as a candidate to induce metastatic colonization. Additional studies using CXCL5 recombinant protein suggest that CXCL5 is sufficient to promote breast cancer cell proliferation and colonization in bone, while inhibition of its receptor CXCR2 with an antagonist blocks proliferation of metastatic cancer cells. This study suggests that CXCL5 and CXCR2 inhibitors may have efficacy in treating metastatic bone tumors dependent on the CXCL5/CXCR2 axis.

## Abstract (web summary)

Breast cancer cells often invade the bone tissue and remain dormant until they are induced to colonize the metastatic site. Here the authors develop an ex vivo co-culture system consisting of bone and cancer cells from mice and show that CXCL5 has a role in metastatic colonization in the bone.

## Introduction

In breast cancer patients with metastatic disease, bone is the most common site of metastasis and the most common site of first distant relapse, with roughly half (48%) of breast cancer patients developing bone metastases after systemic treatment^1–3^. At death, roughly 73% of women with breast cancer^4,5^ have bone metastasis, mostly growing in highly vascularized bones^6^. Breast cancer patients who develop bone metastasis within 4 months of diagnosis have significantly reduced overall survival compared to patients who develop bone metastasis later^7^. While breast cancer patients with metastases exclusively in bone have higher survival rates than patients with metastases in multiple tissues including bone, patients with the worst prognosis have metastasis in multiple tissue locations^8,9^. However, breast cancer metastasis to bone is a significant predictor of recurrence, distant metastasis, and cancer-related death^10,11^.

The risks and complications of bone metastasis are due to not only the bone malignancies themselves but also the resulting tissue destruction. Bone metastasis patients often suffer from severe cancer-induced bone pain, pathological bone fractures, spinal cord compression, aberrant hematopoiesis, cachexia, and hypercalcemia, which can progress into further complications, such as comas^12–14^.

Current treatments for patients with metastatic bone cancer include combinations of chemotherapy, palliative radiation, antiresorptive agents such as bisphosphonates, antibodies that inhibit osteoclastogenesis, and rarely surgical resection^15–17^. Bisphosphonates, particularly zoledronic acid, inhibit osteoclasts and are commonly used to treat hypercalcemia, skeletal-related events, and bone metastasis. Treatment with zoledronic acid increases overall survival in breast cancer patients^18^. Unfortunately, high doses of zoledronic acid cause other adverse events, such as osteonecrosis of the jaw and femoral shaft fractures^19^. Denosumab is a monoclonal antibody that inhibits osteoclast development. Denosumab treatment significantly reduces but does not prevent additional skeletal-related events in patients with breast cancer metastasized to bone^20^ and is also associated with increased risk of osteonecrosis of the jaw^21^. These and related therapies are effective in some patients with bone metastasis, particularly those with bone-only metastasis. However, none are curative in patients with bone metastasis and metastasis to other tissues^22^. Unfortunately, bone metastasis remains incurable with current therapies^13^.

The cells and architecture of the bone provide cancer cells with a fertile soil to harbor circulating tumor cells (CTCs) and to induce them into disseminated tumor cells (DTCs) before colonization in the bone and marrow^23–25^. By releasing cytokines, chemokines, and growth factors, the bone microenvironment inhibits colonization of cancer cells in healthy bone or supports colonization in cancer conditions^26–28^. The bone architecture and the ubiquity of vascular sinusoids provide accessibility to and easy exit from bone to increase the spread through the body from the metastatic bone tumor^29,30^.

Tumor cells and the bone microenvironment engage in signaling crosstalk to form a vicious cycle supporting tumor growth and bone destruction^31–33^. Breast cancer metastasis can begin early, initiated when the primary tumor is too small to detect^34,35^. Then, during cancer progression, CTCs extravasate into other tissues, including bone. However, most cancer cells that escape the primary tumor and travel to another tissue do not form metastatic tumors but instead die or remain dormant, making metastasis inefficient^36–38^. Only a subset of cells both survive in other tissue microenvironments and arrest in a quiescent state called dormancy, which is a major mechanism of resistance to chemotherapy and cancer relapse^39^. Metastatic dormancy is defined by DTCs that survive at the metastatic site and remain quiescent but metabolically active after escaping from the cell cycle, do not divide, and do not grow^40,41^. However, these dormant cells can awake to colonize their new microenvironment. Unfortunately, dormant disseminated cancer cells often are undetected in patients but escape cytotoxic treatment, providing a rationale for late recurrence.

Inducing cancer cell colonization at the metastatic site is considered the rate-limiting step of metastasis but is not well understood^40,42^. Molecular and cellular mechanisms awaken a subset of dormant cells to become proliferative and invasive, thereby promoting cancer cell colonization and growth of metastatic tumors^42,43^. This process can take years or decades^40,42^. Molecular players such as transforming growth factor-β^44^, osteopontin^45^, RON kinase^46^, and C-X-C chemokine motif receptor 4 (CXCR4)^47^ contribute to breast cancer metastasis to bone and to the angiogenic switch that contributes to the escape from dormancy^25,42^. However, comparatively little has been studied about the factors present in the microenvironment niche or the paracrine signaling that may contribute to colonization in intact bone. Understanding what regulates cancer cell colonization is essential to improving the survival and quality of life of cancer patients. To address this problem, we developed an ex vivo culture system in which cancer cells survive, remain metabolically active, and are supported by bone marrow to identify factors, including C-X-C chemokine motif ligand 5 (CXCL5), that regulate the colonization of breast cancer cells metastasized to bone.

## Results

### Mouse bone in culture is viable and supports marrow survival

**Fig. 1:**
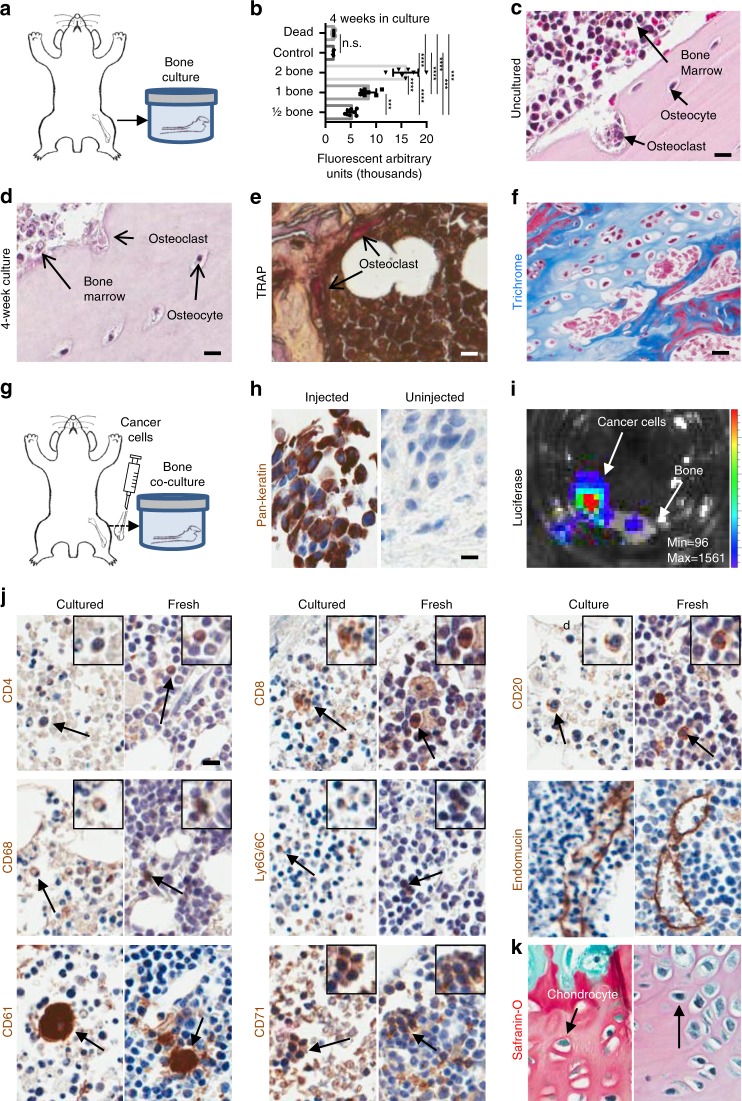
Bone, marrow, and cancer cells survive in ex vivo co-cultures. **a** Experimental design of ex vivo bone cultures using mouse bone explants. **b** Metabolic activity of ex vivo bone cultures compared to the metabolic activity of a dead bone and media controls. CellTiter-Blue (cell viability) assay to test metabolic activity of an increasing number of mouse bone explants in ex vivo culture after 4 weeks in culture (*p* values obtained by Student’s *t* test, multiple comparisons). Lines show the mean and standard deviation. **c** H&E of a fresh, uncultured mouse bone sample (scale bar: 10 μm). **d** H&E of a cultured mouse bone cultured for 4 weeks (scale bar: 10 μm). **e** TRAP staining of a 4-week cultured mouse bone sample. Arrows show the localization of osteoclasts (scale bar: 10 μm). **f** Masson’s trichrome staining on a mouse bone sample after 4 weeks in culture showing retention of collagen depositions shown in blue (scale bar: 20 μm). **g** Experimental design of ex vivo mouse bone co-cultures grown with breast mammary epithelial cancer cells injected into the bone prior to culture. **h** Immunohistochemistry (IHC) for Pan-cytokeratin on a mouse bone co-cultured for 4 weeks with PyMT cancer cells (left) and an uninjected mouse bone cultured for 4 weeks (right). **i** Luciferase assay on a mouse bone sample colonized by a luciferase-expressing PyMT cell line. The intensity bars (rainbow) and scale information (Min/Max) for BLI signal are provided. Bioluminescent PyMT cancer cells co-localize with the bone in culture. **j** IHC of mouse bone marrow cells after co-culture with mammary epithelial cancer cells (left) compared to a fresh mouse bone sample (right) stained for CD4+ helper T cells, CD8+ cytotoxic T cells, CD20+ B cells, CD68+ macrophages, Ly6G/6C+ neutrophils, endomucin endothelial cells, CD61+ megakaryocytes, CD71+ erythroid precursors, and **k** Safranin-O staining of a mouse bone post 4 weeks in co-culture (scale bar: 10 μm). ***Significant at *p* < 0.001; ****significant at *p* < 0.0001; n.s. not significant

To identify factors that induce metastatic colonization, we developed ex vivo bone culture conditions to support survival and growth of bone and cancer cells in culture. First, we identified growth conditions that support bone and marrow viability for 4 weeks (Fig. 1a). To measure bone viability in culture, we quantified the metabolic activity of increasing numbers of cultured mouse long bones (femurs, tibiae, vertebrae) per well (Fig. 1b, Supplementary Fig. 1) and found that bones remained metabolically active after 4 weeks in culture.

To compare the metabolic activity of these cultures to fresh bones and over time, we similarly evaluated metabolic activity of our ex vivo bone cultures in a time course (Supplementary Fig. 1). Interestingly, the metabolic activity of the cultures increased over time, demonstrating that the culture conditions support metabolism. We also included metabolic studies using devitalized dead bone cultures. After the freeze/thaw cycles, the bone and marrow of the dead bone samples had no metabolic activity and were comparable to the fresh media samples grown without bone.

We examined the pathology and expression of resident marrow cell differentiation markers in the cells that survived in bone cultures. The bone pathology detected cells normally found in the bone microenvironment, including osteocytes, osteoclasts, and marrow cells (Fig. 1c, d). Active osteoclasts and collagen deposition were maintained in culture and visualized by hematoxylin and eosin (H&E), tartrate-resistant acid phosphatase (TRAP), and trichrome staining (Fig. 1d–f).

### Cancer cells grow within cultured bone

We co-cultured cancer cells in our bone cultures by injecting cancer cells (mouse PyMT) into the marrow of collected femurs and tibiae from healthy adult mice (Fig. 1g). The co-cultures were grown in low-binding cell culture growth conditions to enrich for epithelial cells that adhere to bone.

The bone cultures supported cancer cells of epithelial origin, as detected by immunohistochemistry (IHC) staining of tissues for the epithelial cell marker Pan-cytokeratin (Fig. 1h, which is not expressed by bone or marrow cells. In contrast, bones grown in the absence of cancer cells did not express Pan-cytokeratin. Therefore, our bone and cancer cell co-cultures support epithelial cancer cell growth at the site of cancer cell injection in the marrow, on the surface of the bone, and in the marrow cavity.

To test survival of the luciferase-expressing cancer cells within the cultures, the metabolic activity of the explants was assayed by luciferase reporter gene detection from the cancer cells. Cancer cells not only were alive and metabolically active but also co-localized with the bones (Fig. 1i).

We determined which immune cells survived 4 weeks in bone cultures by IHC of differentiation markers in bones co-cultured with cancer cells for up to 4 weeks. The marker expression was compared to the expression in fresh, uncultured murine bones. The detected markers included CD4 (cytotoxic T cells), CD8 (helper T cells), CD20 (B cells), CD68 (macrophages), Ly6G/6C (neutrophils), CD61 (megakaryocytes), and CD71 (erythroid precursors) (Fig. 1j). This suggests that hematopoietic lineage cells remain in the marrow cavity in culture. Both cultured and fresh bone samples contained chondrocytes and cartilage by Safranin-O staining (Fig. 1k). Also, both fresh bone and ex vivo cultures expressed endomucin, which stains endothelial cells, demonstrating that ex vivo bone cultures maintain their microvascular structure after weeks in culture (Fig. 1j). Together, the staining demonstrates that the bone co-cultures support growth and viability of bone, marrow, and cancer cells.

### Cancer-primed mouse bone promotes cancer cell proliferation

Metastatic tumors survive and grow in the bone microenvironment while overcoming normal tissue homeostasis. A small fraction of DTCs can remain metabolically active in the marrow cavity for prolonged periods of time without remaining highly proliferative (Ki67 negative; Ki67−) or developing metastases but could eventually become highly proliferative (Ki67 positive; Ki67+) and colonize distant tissues^36,48^. To evaluate cancer cells in our ex vivo bone metastasis cultures for their proliferative potential, we analyzed the proliferation status of the cancer cells in tissue sections by co-staining co-culture tissue samples for Ki67 and Pan-cytokeratin. We ascertained whether the cancer cells (Pan-cytokeratin+) were highly proliferative colonizing cells (Ki67+/Keratin+) or low/non-proliferative quiescent cells (Ki67−/Keratin+) during co-culture (Fig. 1h).

**Fig. 2:**
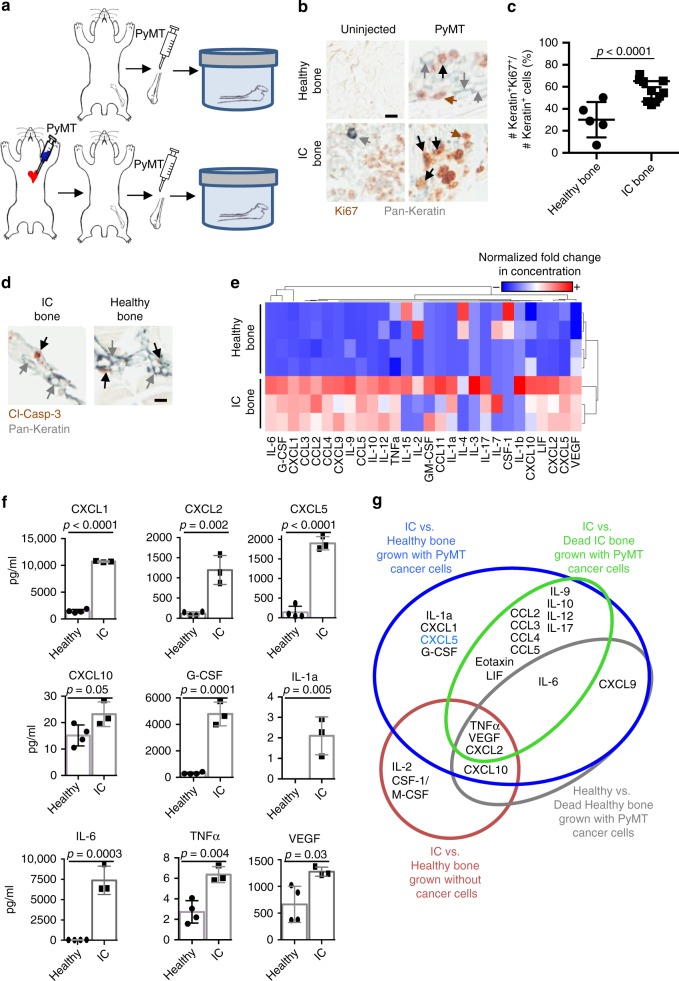
Cancer-primed bones promote cancer cell proliferation. **a** Experimental design of ex vivo bone culture of healthy (top) mouse bone explants and cancer-primed (bottom) bone explants after intracardiac (IC) injection of PyMT cancer cells prior to culture. **b** IHC co-staining for Pan-cytokeratin (gray) and Ki67 (brown) of uninjected or PyMT-injected bone cultures of healthy mouse bone or IC mouse bone co-cultured for 2 weeks. Gray arrows indicate Keratin+Ki67– cells, brown arrows indicate Ki67+ only cells, and black arrows indicate Keratin+Ki67+ cells (scale bar: 10 μm). **c** Comparison of percentage of Ki67+ cancer cells over total number of cancer cells of mouse bone between the healthy and the IC bone co-cultures after a 2-week culture period (*p* < 0.0001, Student’s *t* test). Each dot represents the percentage of Ki67+Keratin+ cancer cells detected in one section of the bone. Lines show the mean and standard deviation. **d** IHC co-staining for Pan-cytokeratin (gray) and cleaved-caspase 3 (apoptosis marker). Neither IC nor healthy bone co-cultures present a high number of dying cells positive for cleaved-caspase 3. Gray arrows indicate Keratin+Ki67– cells, and black arrows indicate Keratin+cleaved-caspase 3+ cells (scale bar: 10 μm). **e** Heatmap of quantified chemokines and cytokines demonstrating unique protein expression profiles in the media supernatant of healthy bone and IC bone co-cultures with PyMT cancer cells. Higher concentrations of chemokines are shown in red and lower concentrations are shown in blue. Values in the heatmap show normalized fold increase concentration for each soluble factor. **f** Quantification of cytokine and chemokine protein concentration in conditioned media from co-cultures grown for 2 weeks with healthy bone or IC bone in co-culture with PyMT cells. Cytokines and chemokines included: CXCL1 (*p* < 0.0001), CXCL2 (*p* = 0.002), CXCL5 (*p* < 0.0001), CXCL10 (*p* = 0.05), G-CSF (*p* = 0.0001), IL-1a (*p* = 0.005), IL-6 (*p* = 0.0003), TNFα (*p* = 0.004), and VEGF (*p* = 0.03). All *p* values were generated by Student’s *t* test. **g** Venn diagram illustrating the cytokines and chemokines differentially expressed in the media supernatant of dead, healthy, and IC bone culture with or without cancer cells

We compared the proliferative potential of co-cultures grown with healthy noncancerous bones to cultures grown with bones from animals that already support cancer cell growth (Fig. 2a). The cancer-bearing animals were generated by priming the animals with PyMT cancer cells by intracardiac (IC) injection into normal adult FVB/N mice (Fig. 1g). Then IC-primed and healthy bones were injected with PyMT cells and co-cultured for 2 weeks.

Alternatively, as a control sample, additional cultures of the mock-injected IC-primed bones (uninjected with cancer cells) were used to determine whether the cancer cells seen in culture were directly derived from the IC-injected cancer cells or from the cancer cells injected into the cultured bone. After 2 weeks, the cultured bones were stained for Pan-cytokeratin and Ki67, and the presence of PyMT cells and their proliferation status was measured. The bones cultured without cancer cells gave rise to, at most, a small number of single Keratin+ cancer cells that were not proliferating (Ki67−; Fig. 2b). Therefore, the cancer cells growing in the IC-primed bone cultures were most likely added ex vivo. However, even though we could not detect many cancer cells in the bones collected after IC injection, we cannot rule out the possibility that some cancer cells detected in co-cultures may be derived from the original IC injection.

To compare the proliferation status of the cancer cells grown in IC-primed and healthy bone cultures, histological sections of bone samples were co-stained for Pan-cytokeratin and Ki67 by IHC (Fig. 2b). Interestingly, quantification of the Ki67+Keratin+ cells showed that the IC bone co-cultures had a significantly higher percentage of Ki67+Keratin+ cells than was seen in the healthy bone co-cultures (Fig. 2c).

Quiescent cancer cells remain metabolically active, even though they are not proliferating. However, the metabolic activity of a culture is not necessarily stagnant, particularly since cell cultures are heterogeneous and include a population of cells with stem cell properties that may include both quiescent cells as well as some proliferative cells. Some cells, including stem cells, reversibly switch between quiescence and proliferation^49^. The metabolic states also change; stem cells maintain high glycolytic rates but low oxygen consumption^50,51^.

To test for metabolic activity of the cancer cells in our co-cultures, we injected a range of PyMT cancer cell numbers expressing luciferase into either healthy or IC-primed mouse bones and quantified luciferase activity of the PyMT cancer cell reporter gene by weekly addition of D-Luciferin and imaging the bioluminescence produced by the cancer cells. Cancer cells from both the healthy bone co-cultures and the IC-primed bone cultures expressed luciferase (Supplementary Fig. 2). These data support metabolic activity in both healthy and IC-primed bone cultures.

These data together demonstrate that cancer cells grown in IC-primed bones are more proliferative and metabolically active than cancer cells grown in healthy bones. Therefore, the bone microenvironment of the IC-injected animals was primed to better support cancer cell proliferation than the bone microenvironment from normal animals.

### Apoptosis does not account for the changes in proliferation

To determine whether the low number of proliferating cells in healthy bones was caused by an increased number of dying cells, we co-stained tissues by IHC for Pan-cytokeratin and cleaved-caspase 3 (Fig. 2d). Very small numbers of Keratin+ cancer cells were positive for cleaved-caspase 3 in either of the culture conditions. These results suggest that apoptosis does not account for the decreased number of proliferating cancer cells that we observed in healthy bone cultures.

### CXCL5 is a candidate regulator of bone colonization

Cytokines, chemokines, and growth factors are soluble mediators secreted from the marrow that regulate angiogenesis, cellular growth control, cellular motility, wound healing, and inflammatory responses via paracrine signaling with neighboring cells. These factors have been characterized in the immune system by their pro-inflammatory and chemoattractant properties^52,53^ but recently were  identified as mediators of tumor progression^54–56^. We screened the secreted cytokines and chemokines in the conditioned media samples of our cultures (Fig. 2e). Interestingly, co-cultures of bones grown with cancer cells secreted significantly more factors into the media than cultures grown without cancer cells. These factors could be from paracrine signaling or from the cancer cells themselves.

We wanted to identify factors enriched in the IC-primed bones that potentially regulate paracrine signaling between the cancer cells and the IC bone microenvironment to promote cancer cell proliferation and colonization of bone. To do this, first we compared the factors found in the conditioned media from IC-primed bone and healthy bone co-cultures grown with and without cancer cells (Fig. 2f). The analysis of the conditioned media identified proteins differentially expressed between healthy and IC-primed bone cultures, as determined by hierarchical clustering analysis and represented by heatmap (Fig. 2e). The differentially expressed factors produced exclusively in cultures with cancer cells included interleukin (IL)-1a, CXCL1, CXCL5/LIX, granulocyte colony-stimulating factor (G-CSF), IL-6, leukemia inhibitory factor, C-C chemokine ligand 2 (CCL2)/monocyte chemotactic protein-1, and CCL4/macrophage inflammatory protein (MIP)-1B, and the factors produced in cultures without cancer cells included IL-2, CSF-1/macrophage CSF, tumor necrosis factor-α (TNFα), vascular endothelial growth factor (VEGF), CXCL2/MIP2, and CXCL10 (Fig. 2g).

To exclude factors produced by healthy bones that contribute to regular bone turnover, we identified factors expressed by healthy bone and marrow in our culture conditions by comparing the conditioned media from healthy bone cultures and devitalized healthy cultures in which the bone went through multiple freeze/thaw cycles to kill all bone and marrow cells (dead bone). Factors that distinguished between these samples were likely secreted factors produced or regulated by healthy bone and include TNFα, VEGF, CXCL2, CXCL9, CXCL10, and IL-6.

We separated the remaining factors produced by the IC-primed bone cultures into those that were or were not dependent on bone and marrow by including or not the factors produced by IC-primed vs. dead IC-primed bone cultures. Factors that differed significantly between IC-primed and the dead IC-primed bone cultures were likely factors secreted from the bone or marrow of IC bone and are potential important factors required for paracrine signaling with the cancer cells. The four factors that clearly distinguished IC-primed bone from healthy bone but do not differ between IC-primed and dead IC-primed bone cultures were CXCL1, CXCL5, G-CSF, and IL-1a.

A secreted factor that regulates the balance between quiescence and proliferation could activate and be required for promoting proliferation or, alternatively, could inhibit proliferation by actively suppressing proliferation or inducing quiescence. Among the factors identified in our screening, a panel of cytokines and chemokines identified as neutrophil homeostasis regulators (IL-17, G-CSF, and CXCL5) was also differentially found in our co-cultures^57^. CXCL5 was of particular interest because the CXCL5 concentration was higher in conditioned media from cancer-primed bones, which were proliferative, compared to normal healthy bones, which were less proliferative (Fig. 2e, f).

### CXCL5 protein is detected in cancer cells and marrow

CXCL5 may promote proliferation by activating a signaling cascade. CXCL5 and its only known functional receptor CXCR2 play a role in promoting angiogenesis, migration, invasion, local recruitment of neutrophils, and poor response to chemotherapies^54–56,58–60^ as well as with cancer progression and metastasis in breast, prostate, liver, bladder, and colorectal cancers^54–56,59,60^. In contrast, the contribution of the CXCL5/CXCR2 axis to breast cancer colonization in bone is unexplored.

**Fig. 3:**
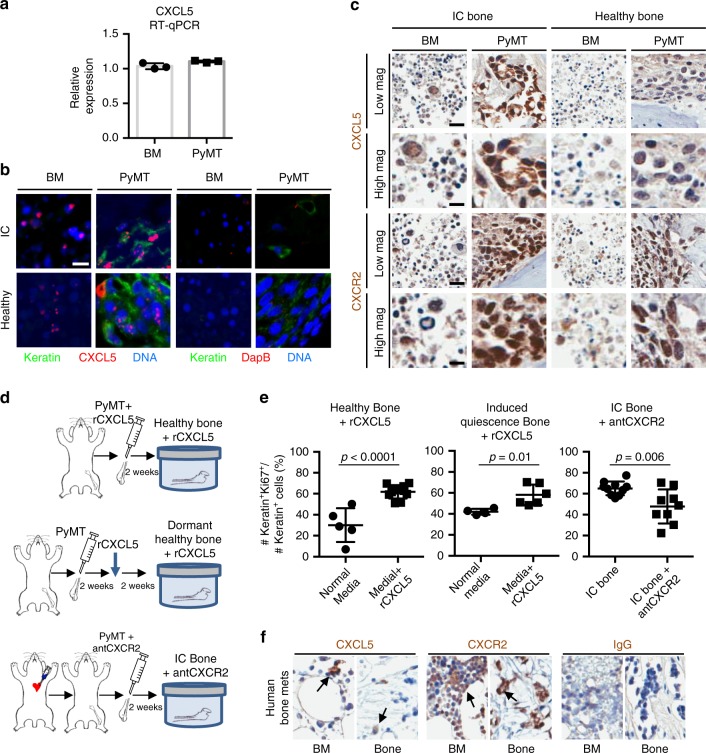
CXCL5 and CXCR2 contribute to the regulation of proliferation. **a** RT-qPCR analysis for CXCL5 expression of bone marrow of healthy mice and PyMT cancer cells. **b** In situ hybridization (ISH) analysis of paraffin sections of healthy or IC mouse bone co-cultured for 2 weeks with PyMT cells. ISH fluorescent probe (red) was used to detect RNA transcripts in the bone marrow and in Keratin+ cancer cells (green). DapB ISH probe was used as a negative control (scale bar: 10 μm). **c** IHC staining for CXCL5 and CXCR2 of healthy and IC bone co-cultures injected with PyMT cells (scale bars: Low magnification = 20 μm; High magnification = 10 μm). **d** Experimental design of ex vivo bone co-cultures. (Top) Healthy mouse bone explants supplemented daily with rCXCL5 protein. (Middle) Healthy mouse bone explants cultured for 2 weeks to achieve a low-proliferative phenotype of cancer cells. These cultures were subsequently supplemented daily with rCXCL5 protein for the next 2 weeks. (Bottom) Cancer-primed bone explants using bones from animals with intracardiac (IC) injection of PyMT cancer cells prior to culture and supplemented daily with CXCR2 antagonist SB225002. **e** Percentage of Ki67+Keratin+ cancer cells over total number of Keratin+ cancer cells of mouse bone co-cultures (Left) between healthy bone with normal media and healthy bone with rCXCL5 complemented media (*p* < 0.0001, Student’s *t* test), (Middle) between healthy bone with normal media and healthy bone with rCXCL5 complemented media 2 weeks after start of co-culture (*p* = 0.01, Student’s *t* test), and (Right) between cancer-primed bone with normal media and cancer-primed bone with a CXCR2 antagonist complemented in the media (*p* = 0.006, Student’s *t* test). Each dot represents the percentage of Ki67+Keratin+ cancer cells detected in one section of bone. Lines show the mean and standard deviation. **f** IHC staining for CXCL5 and CXCR2 of a metastatic human femoral head from a patient with breast cancer metastasis to bone. CXCL5 and CXCR2 were found in the human bone samples (scale bar: 20 μm)

We analyzed CXCL5 expression by reverse transcription quantitative polymerase chain reaction (RT-qPCR) of cell lysates as well as by in situ hybridization (ISH) and IHC of these cells in culture. First, to determine which cells produce CXCL5, we conducted RT-qPCR analysis on freshly collected bone marrow and PyMT cancer cells (Fig. 3a). CXCL5 expression was detected in mouse marrow and in PyMT cells.

We analyzed CXCL5 gene expression in our bone co-cultures by fluorescence ISH. ISH detected CXCL5 expression in both Keratin+ and Keratin− cells in both healthy and IC bone cultures (Fig. 3b). Therefore, both cancer cells and cells normally present in the marrow express CXCL5 when co-cultured.

We next analyzed CXCL5 and CXCR2 protein expression by IHC of CXCL5 in bone tissue sections from healthy and IC bone cultures (Fig. 3c). Similar to the gene expression data, CXCL5 protein was detected not only in marrow cells but also in cancer cells. Together, CXCL5 protein is expressed by marrow and cancer cells before and during the culture period. Both the cancer cells and additional marrow cells expressed CXCR2 protein, including bone cells with the pathology of osteocytes and chondrocytes (Fig. 3c and Supplementary Fig. 2). These data support CXCL5 and CXCR2 protein expression in both the cancer cells and in other cells located within the marrow cavity.

Interestingly, CXCL5 and CXCR2 protein expression by IHC staining appears more easily detected in cancer cells and bone marrow of IC bone cultures compared to healthy bone cultures and could reflect a higher CXCL5 and CXCR2 protein expression in these cells within IC bone co-cultures (Fig. 3c). This increased expression could contribute to the increased proliferation by activation of the CXCL5/CXCR2 axis.

### The CXCL5/CXCR2 axis promotes bone colonization

We determined whether CXCL5 is necessary and sufficient to promote cancer cell proliferation and colonization in bone. To test this, we added exogenous recombinant CXCL5 protein (rCXCL5) to healthy bone cultures or SB225002, an inhibitor of CXCR2^61^, to our highly proliferative IC bone cultures and quantified the percentage of Ki67+Keratin+ cancer cells (Fig. 3d).

We added rCXCL5 to healthy cultures under two conditions: (1) at the start of a fresh culture, which models CXCL5 as a mechanism to maintain or promote proliferation, and (2) after the culture was in culture long enough to induce quiescence (Ki67−), to model overcoming quiescence and initiating colonization. The CXCL5 concentration was selected from the conditioned media CXCL5 concentration (Fig. 2f). We stained the samples for Ki67 and Keratin to check the cancer cell proliferation with the addition of CXCL5. Indeed, addition of rCXCL5 to the cultures significantly increased the Keratin+Ki67+ cells, suggesting that CXCL5 is sufficient to promote cancer cell proliferation (Fig. 3e, left).

Furthermore, addition of rCXCL5 for 2 weeks to the media of samples that had already been in co-culture for 2 weeks helped recover proliferation in cancer cells (Fig. 3e, middle). This suggests that addition of rCXCL5 to the co-cultures at the beginning of the culture overcomes any reduced proliferation and recovers/reactivates proliferation if added after the cancer cells reach their low proliferative state.

If blocking CXCL5’s receptor CXCR2 is detrimental to the proliferative state of the cancer cells in dead bone cultures, then blocking CXCR2 with the selective non-peptide CXCR2 antagonist SB225002 (antCXCR2) should attenuate CXCL5 activity by inhibiting CXCR2 and reduce cancer cell proliferation. Curiously, antCXCR2 reportedly binds to multiple epitopes within the CXCR2 receptor, but these inhibitory sites do not overlap with the binding site of agonists within the same receptor and instead are allosteric inhibitors of CXCR2 activity^62^.

We added antCXCR2 to the culture media of IC-primed bone cultures, which support cancer cell proliferation, to determine whether CXCR2 inhibition was sufficient to inhibit cancer cell proliferation. Cancer cell proliferation significantly decreased compared to controls after antCXCR2 addition, suggesting that CXCR2 is necessary to induce proliferation in the IC-primed bone cultures (Fig. 3e, right).

Our results demonstrate that CXCL5 is sufficient to induce cancer cell proliferation in bone cultures and that blocking CXCR2 with an antagonist inhibits proliferation of cancer cells and prevents CXCL5-induced signaling. These results are consistent with CXCL5 acting through its receptor CXCR2 to induce cancer cell proliferation (Fig. 3e).

### Human bone metastasis expresses CXCL5 and CXCR2 protein

We stained human femoral head tissue sections from a patient with breast cancer metastasis to bone for CXCL5 and CXCR2 by IHC. CXCL5 and CXCR2 stained both bone and marrow cells. CXCL5-positive cells were commonly found in small cell clusters, while CXCR2 was more ubiquitously detected (Fig. 3f). Therefore, the CXCL5/CXCR2 axis may be physiologically relevant to human patients affected by metastatic disease to the bones from breast cancer.

### Healthy bone induces cancer cell quiescence in co-culture

**Fig. 4:**
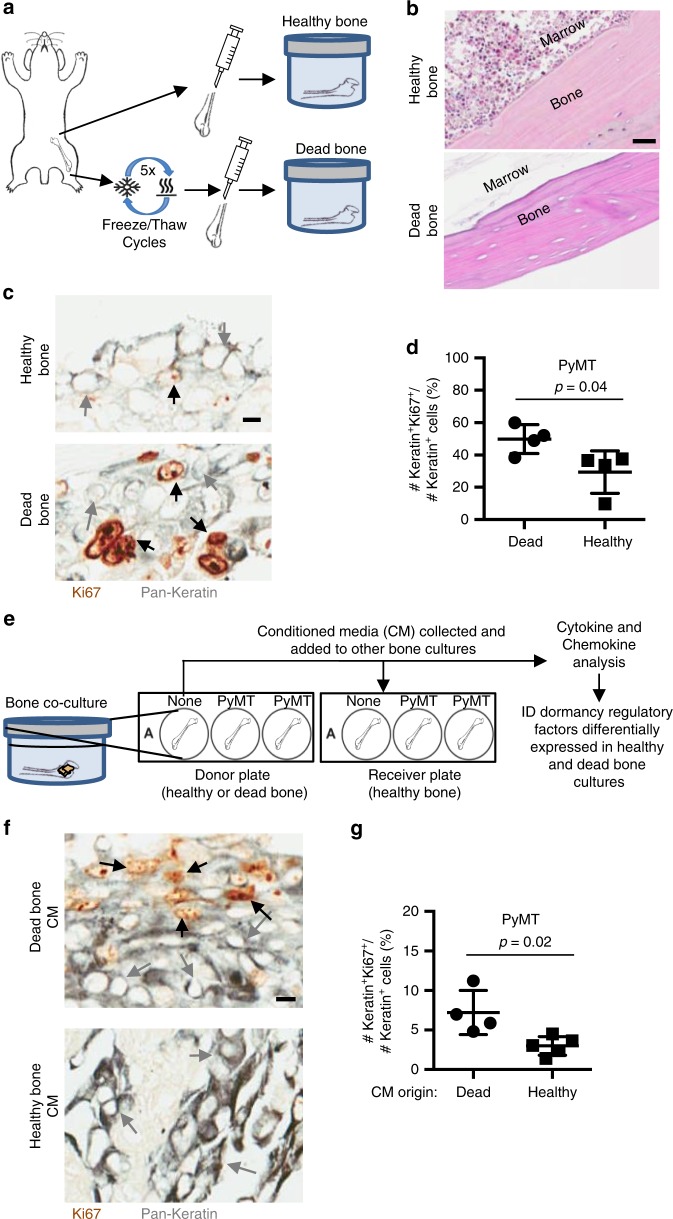
Healthy mouse bones inhibit cancer cell proliferation. **a** Experimental design of healthy (live) and dead bone co-cultures grown with breast mammary epithelial cancer cells. Dead bone was defined as healthy bone explants that underwent at least five freeze/thaw cycles prior to culture. **b** H&E staining of a healthy bone (top) and a dead bone (bottom) explant to show the devitalized bone lacking viable cells in bone and marrow (scale bar: 30 μm). **c** IHC co-staining for pan-cytokeratin (gray) and Ki67 (brown) in healthy (top) and dead bone (bottom) co-cultures after 3 weeks in culture. Gray arrows indicate Keratin+Ki67– cells, and black arrows indicate Keratin+Ki67+ cells (scale bar: 10 μm). **d** Comparison of percentage of Ki67+Keratin+ cancer cells over total number of Keratin+ cancer cells of mouse bone in healthy and dead bone co-cultures cultured with PyMT cells (*p* = 0.04). **e** Experimental design of co-cultures with conditioned media added to co-cultures of healthy bone and breast mammary epithelial cancer cells. Conditioned media was collected from previous cultures of healthy or dead bone co-cultures and either added to additional co-cultures in combination with fresh media for the new co-cultures or was sent for analysis. **f** IHC co-staining for pan-cytokeratin (gray) and Ki67 (brown) of a 2-week co-culture of healthy bone samples with cancer cells using conditioned media from (top) dead bone or (bottom) healthy bone. Gray arrows indicate Keratin+Ki67– cells, and black arrows indicate Keratin+Ki67+ cells (scale bar: 10 μm). **g** Comparison of percentage of Ki67+Keratin+ cancer cells over total number of Keratin+ cancer cells of mouse bone in healthy and dead conditioned media bone co-cultures with PyMT cells (*p* = 0.02). For all Keratin and Ki67 staining comparison, each dot represents the percentage of Ki67+Keratin+ cancer cells detected in one section of bone. Lines show the mean and standard deviation. All *p* values were calculated by Student’s *t* test

We next wanted to investigate whether a healthy bone microenvironment provides a quiescence-inducing effect on its own by inhibiting proliferation. To address this, we co-cultured cancer cells with healthy bone freshly extracted from animals (healthy bone) or with devitalized bone generated after at least five freeze/thaw cycles prior to culture (dead bone) (Fig. 4a). The dead bone cultures afforded the cancer cells a bone surface to attach to for anchorage-dependent growth but without intact molecular factors released by the bone microenvironment to participate in paracrine signaling with the cancer cells. The dead bone tissue samples showed no live osteocytes or marrow cells but still preserved the bone matrix by histological analysis (Fig. 4b).

Interestingly, the Ki67+ expression status was higher in dead bone cultures than in healthy bone cultures, indicating high proliferation in the dead bone cultures and low proliferation in the healthy cultures (Fig. 4c, d). These results suggest that a factor from the bone or marrow microenvironment actively regulates the balance between proliferation and quiescence in healthy bone co-cultures.

### Quiescent cell conditioned media inhibits proliferation

Our data suggest that cancer cell quiescence can be regulated by a factor produced from the bone microenvironment by paracrine signaling and could be initiated by signals from the osteocytes in the bone, bone marrow, or the culture media itself.

To determine whether a factor is secreted from the bone/marrow in our healthy bone co-cultures, we tested the ability of conditioned media from healthy or dead bone cultures for their ability to support cancer cell proliferation in healthy bone culture conditions, which normally support reduced cancer cell proliferation. First, we collected mouse bones and used them for dead or healthy co-cultures. After 2 weeks in culture, the conditioned media was collected from and was used daily to supplement another cohort of healthy bone co-cultures (Fig. 4e). The cancer cell co-cultures grown with conditioned media from healthy bone cultures had lower proliferation rates than co-cultures grown with conditioned media from dead bones (Fig. 4f, g). These results suggest that healthy bone cultures release factors into the media that induce quiescence or inhibit the proliferation of cancer cells in culture.

To further investigate the differences between these cultures, we compared the chemokine and cytokine expression profiles from the conditioned media. CXCL5 levels in the conditioned media from healthy and dead healthy bone cultures were not different (Fig. 2g). This suggests that a factor other than CXCL5 accounts for the proliferative differences between these two cultures. The expression analysis of these samples identified IL-6, TNFα, VEGF, CXCL2, CXCL9, and CXCL10 as differentially expressed between healthy and dead bone cultures. All except CXCL9 were produced at higher levels in healthy than in dead bone cultures. These factors might include regulators of cancer cell proliferation in the bone microenvironment or be components of pathways that inhibit cancer cell proliferation in healthy bone.

## Discussion

One of the major obstacles in studying the initiation of metastatic colonization in the bone has been the lack of experimental models available to study this step in cancer progression without the confounding variables of earlier steps of the metastatic cascade. This study developed an ex vivo co-culture methodology that simulates the last stages of cancer metastasis to bone and cancer cell colonization. Our culture conditions maintain a reversible cancer cell quiescent state when grown in healthy bones and maintain proliferative cancer cells when grown in cancer-primed IC bones. Utilizing our co-culture methodology, we identified a panel of chemokines and cytokines that are candidate regulators of the balance between cancer cell quiescence/dormancy and colonization of bone, a process we refer to as the dormancy switch. We identified candidate inducers of both quiescence and colonization including CXCL5 as a regulator of breast cancer quiescence and as sufficient to induce bone metastasis colonization. Also, CXCR2 signaling is required for cancer cell proliferation and colonization in bone. Consistently, cancer-bearing bone cultures secreted more CXCL5 than healthy bone, suggesting either that healthy bone produces an inhibitor of CXCL5 release or that cancer bone produces/releases increased levels of CXCL5.

**Fig. 5:**
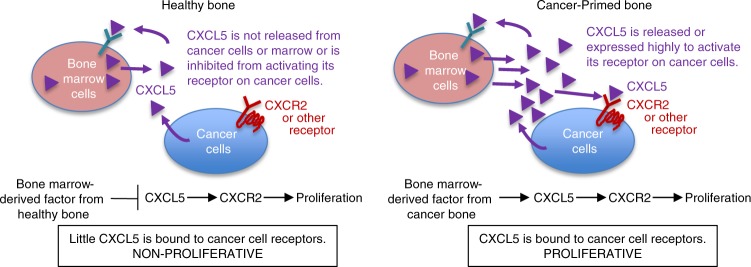
Model of CXCL5/CXCR2 in metastatic bone colonization. Proposed model of the dormancy switch to colonization by CXCL5/CXCR2 axis in cancer cells. Bone marrow cells from healthy bone produce factors that act as sponges for or inhibit CXCL5, making CXCL5 unavailable for binding to its receptor on cancer cells and activating proliferation, thus causing the cancer cells to remain quiescent. In contrast, cancer-primed bone either inhibits or does not produce the factors that inhibit CXCL5. CXCL5 binds and activates its receptor on the cancer cells, ultimately inducing cancer cell proliferation and colonization at the bone metastatic site

Based on our experimental results, we hypothesize that healthy bones in vivo and in co-culture make factors that inhibit cancer cell proliferation, while cancer-primed bones produce factors that promote cancer cell proliferation (Fig. 5). These studies suggest that the soil of the metastatic site can be modified by the initial vanguard of cancer cells prior to the colonization of cancer cells to generate a microenvironment that is hospitable to and supportive of cancer cell proliferation.

Our ex vivo bone co-culture assay preserves the bone cellular heterogeneity and the three-dimensional microenvironment, including heterogeneous cell types found in normal bone and marrow. In contrast to in vivo models, cancer cells in these cultures are grown independently of the primary tumor and after extravasation of the tumor cells into the bone marrow. A major advantage of this methodology is the potential to modify and test multiple culture conditions in parallel in controlled conditions. Testing these conditions has led to our discovery of a unique model system that can be used to investigate the role of additional chemokines like CXCL5 and their receptors in metastatic tumor colonization.

This study suggests that bones primed with cancer cells form a niche that actively supports the proliferation of metastatic cells and is inducible by CXCL5. Previous studies provide a precedent for identification of factors that induce a pre-metastatic niche. The hypoxic secretome forms pre-metastatic lesions that will later be occupied by metastatic cells^63^. These focal pre-metastatic lesions are mediated by lysyl oxidase, a regulator of osteoclastogenesis and bone homeostasis that is independent of receptor activator of nuclear factor-κB ligand^63^. The pre-metastatic bone lesions will be occupied by CTCs and will develop as bone metastases. Hematopoietic progenitor cells that express VEGFR1 also induce the formation of pre-metastatic niches in the bone and marrow^64^. Interestingly, these are consistent with our study, since we also saw a significant increase in VEGF in the conditioned media from IC-primed bone cultures compared to healthy bone cultures. In melanoma, tumor-derived exosomes also support the formation of pre-metastatic niches and tumor growth by educating marrow cells via the receptor tyrosine kinase MET^65^. These studies support the importance of the communication between the bone microenvironment and cancer cells to further promote metastatic colonization.

In culture, devitalized bones and conditioned media from these cultures support cancer cell proliferation, suggesting that the bone and/or marrow cells express an inhibitor of cancer cell proliferation. A candidate inhibitor of CXCL5 released during bone metastasis is ACKR1/DARC. DARC is a promiscuous chemokine decoy/nonfunctional receptor that is expressed highly on the surface of erythrocytes and endothelial cells^66^. DARC acts as a scavenger receptor that can bind to a number of angiogenic chemokines from the CC and CXC chemokine families, including CXCL5, CXCL1, and CXCL2^66,67^. While DARC expression and metastasis are inversely related, the role of DARC in regulating cancer proliferation and initiation of colonization during metastatic progression has not been investigated. Therefore, a possible explanation for our observation is that healthy bone marrow cells express high levels of DARC and act as scavengers for CXCL5, depleting CXCL5 from the media and making CXCL5 inaccessible to cancer cells in co-culture. In contrast, marrow cells in cancer-primed animals either do not express DARC or express excess CXCL5 that can activate CXCR2 expression in the cancer cells.

CXCL5 has been detected in non-immune cells including osteoblasts, endothelial cells, and fibroblastic cells as well as in immune cells, such as neutrophils, monocytes, macrophages, and platelets^54,56,68–70^. Interestingly, our experiments showed CXCL5 expressed not only in the marrow but also in mammary epithelial cancer cells in co-culture and in human metastatic bones. Furthermore, when breast cancer cells are in close proximity to osteoblasts, CXCL5 is produced by the osteoblasts^68^. In the breast cancer cells, CXCL5 then promotes the increased phosphorylation and activation of Raf/mitogen-activated extracellular signal-regulated kinase/extracellular signal–regulated kinase, MSK1, Elk-1, and the transcription factor Snail, which suppresses E-cadherin, leading to an increase in epithelial-to-mesenchymal transition, motility, and migration^56^. Future in vivo validation of CXCL5 actions on breast cancer cells at metastatic bone niches in breast cancer patients with metastatic disease will further support our ex vivo culture findings that led to the identification of CXCL5 as a mechanism of bone colonization.

CXCR2 is more frequently highly expressed in malignant breast tumor cell samples than in benign samples^71^. Similarly, breast cancer cell lines with high CXCR2 expression showed increased invasiveness and metastatic potential compared to cells with CXCR2 knocked down^60^. In highly metastatic breast cancers, blocking CXCR2 decreases chemoresistance and metastasis to the lungs^72^. Furthermore, knocking down CXCR2 in the host significantly reduced tumor growth by reducing angiogenesis, proliferation, and enhancing apoptosis^73^. Future research will be required to determine whether complete removal of CXCR2 is sufficient to overcome CXCL5-induced cancer cell proliferation or whether other CXCL5-bound receptors contribute.

This study suggests that CXCL5 and CXCR2 inhibitors are attractive therapeutic targets that may have efficacy in treating or inhibiting the formation of metastatic bone tumors that are dependent on the CXCL5/CXCR2 signaling axis and resistant to current therapies. Since patients with these tumors currently have few treatment options and often are incurable, this study could have significant translational potential. Importantly, our experimental approach is not only restricted to the study of breast cancer but is also applicable to other cancers that metastasize to bone. Also, in addition to CXCL5, our study identified a panel of additional factors expressed in IC cancer-primed bones that are also candidate regulators of colonization and should be the focus of future studies of breast cancer metastatic colonization of bone.

## Methods

### Mice

The mouse models used in this study included the following transgenic mouse line: FVB. FVB mice were acquired from Harlan Laboratories, Inc. (now Envigo). All experiments in Table 1 used bones from FVB immunocompetent mice.

**Table 1:** Overview of samples used in this study

Bones cultured	Bone type	Time in culture (weeks)	Added to culture	# of bone cultures	# Mice	Total # Keratin+ cells analyzed per sample^a^	# Keratin+Ki67+/Total # Keratin+ cells per sample (%)	Compared to	*p* Value (*t* test)	Purpose of samples
Live	Healthy	2	None	5	4	745	30	IC 2 weeks	<0.0001	Viability
Live	Healthy	3	None	4	3	223	29	Dead 3 weeks	0.04	Viability
Live	Healthy	3	None	4	3	223	29	Live 2 weeks	n.s.	Viability
Live	Healthy	4	None	4	3	1960	42	rCXCL5 2+2	0.01	Viability
Live	Healthy	4	None	4	3	1960	42	Live 2 weeks	n.s.	Viability
Live	IC	2	None	10	4	751	65	Live 2 weeks	0.0001	IC cancer-primed bone
Live	Healthy	2	rCXCL5	11	4	1946	62	Live 2 weeks	<0.0001	CXCL5 sufficiency
Live	Healthy	2+2	rCXCL5	6	4	1310	58	Live 4 weeks	0.01	CXCL5 recovery
Live	IC	2	CXCR2 antagonist	9	4	960	48	IC 2 weeks	0.006	CXCR2 antagonist
Dead	Healthy	3	None	4	2	848	50	Live 3 weeks	0.04	Dead vs. live bone
Live $	Healthy	2	None	5	2	1513	3	Dead CM (@)	0.02	CM
Live @	Healthy	2	None	4	3	1565	7	Live CM (#)	0.02	CM

Samples were cultured with fresh media unless conditioned media is indicated. The bones were generated from wild-type FVB/N mice injected with PyMT cancer cells and incubated for 2 weeks prior to collecting bones for use in the bone cultures. The numbers in the table were rounded to the nearest whole number. The conditioned media was collected from cultures grown with cancer cells. This reflects the culture from which the conditioned media was collected. The Live $ sample grew with conditioned media collected from live, healthy bones. The Live @ sample grew with conditioned media collected from dead bones. 2+2 refers to samples cultured in fresh media for 2 weeks and then cultured for an additional 2 weeks with rCXCL5 added to the media

*#* number, *avg* average, *Dead* bone that went through a series of freeze/thaw cycles, *IC* intracardiac, *Live* live bone used in culture, *rCXCL5* recombinant CXCL5, *CM* conditioned media

^a^The average of the values for the samples analyzed

### Ethical animal usage

Mice used in this study were maintained under pathogen-free conditions in the University of Notre Dame Freimann Life Sciences Center. Animal experiments were conducted with approval from the University of Notre Dame Institutional Animal Care and Use Committee (IACUC) for the ethical treatment of animals (protocol # 15-10-2724).

### Cell line and cell culture

All cancer cell lines and co-cultures were carefully maintained in culture under sterile conditions at 37 °C and grown in 5% CO_2_ using standard culture media containing Dulbecco’s modified Eagle’s medium (DMEM) H21 (Sigma, cat. # D5648) with 10% fetal bovine serum (FBS; JR Scientific, cat. # 43640). For cancer cells, we used a mouse mammary epithelial cancer cell line derived from a bone metastasis from the MMTV-PyMT mouse model (source: Conor Lynch)^74^. These cells were selected for their ability to metastasize to bone. The PyMT cells express a luciferase reporter gene. Our cell line was authenticated prior to the initial experiments using short tandem repeat profiles (Genetica cell line testing), tested negative for mycoplasma contamination using a mycoplasma detection kit (InvivoGen, cat. # rep-pt1), and checked regularly for preservation of their typical morphology and behavior.

### Bone culture

For ex vivo bone cultures used in Table 1, femurs and tibiae were collected from healthy or tumor-bearing FVB/N mice and transplants, cut in half at the mid-diaphysis, cleaned to remove all connective tissue by cutting away the muscle and ligaments around the bone and by using clean gauze to remove the remaining connective tissue attached to the bone, pierced to form a hole at the epiphysis with a 25-gauge needle, and immediately placed into 12-well low-cell-binding plates (Thermo Scientific #145385) with 2 mL of culture media (DMEM H21 with 10% FBS and 1% antibiotics (Sigma #P4458) per well. Cancer cells (2.5 × 10^5^ cells) were resuspended in 10 μL of culture media and carefully injected into the bone marrow through the open end of the diaphysis with a 27-gauge needle. The number of breast cancer cells (2.5 × 10^5^) injected into the mouse bones for ex vivo bone cultures was chosen because it is the number of cells injected into mouse tibiae for in vivo intratibial injections^75^. After injection, the bones grew in culture for 2–4 weeks at 37 °C and 5% CO_2_ in the 12-well low-cell-binding tissue culture plates.

Half of the culture media from each bone culture was replaced daily with fresh media to supply nutrients for the bone, marrow, and cancer cells. Half of the original media, or conditioned media, remained in each well to maintain proteins and other factors secreted into the media by the bone, marrow, and cancer cells. After culturing for the indicated time points, the bone samples were fixed overnight in 4% paraformaldehyde (PFA) at 4 °C, and stored the following day in 70% ethanol.

For conditioned media experiments, half of the culture media from each bone culture was replaced daily with a mixture of a 1:1 ratio of fresh media and conditioned media from a donor plate.

For rCXCL5 and antCXCR2 experiments, half of the media was replaced daily with fresh media adding either rCXCL5 (2 ng mL^−1^ final concentration per well) or the CXCR2 antagonist SB225002 (100 nM final concentration per well). SB225002 is a potent and selective nonpeptide inhibitor and antagonist of CXCR2^61^. The rCXCL5 and SB225002 were added daily during the media change. For the experiment testing recovery of proliferation by addition of rCXCL5 after loss of proliferation, samples were co-cultured for 2 weeks with normal media, and CXCL5 was added daily to the complemented media for the following 2 weeks. The total co-culture time was 4 weeks.

The concentration of CXCL5 was selected because it was approximately the concentration of CXCL5 detected in the conditioned media from IC-primed bones (1.75 ng mL^−1^) (Fig. 2f). We used the CXCR2 antagonist SB225002 at a final concentration of 100 nM in each well based on the IC50 value of SB225002 (22 nM) and on previously determined inhibition of CXCR2 activity at 100 nM^61^.

### IC injections

On the day of injection, cancer cells were trypsinized, collected, and resuspended at 1 × 10^6^ cells mL^−1^ in ice-cold phosphate-buffered saline (PBS; without calcium or magnesium). After the mouse was anesthetized, it was in the supine position and the abdominal hair was removed with an electric razor followed by cleaning the shaved area with an alcohol swab. Using a 27-gauge needle, we injected 100 μL of the cancer cell suspension (1 × 10^5^ cancer cells per animal) into the left cardiac ventricle of the mouse (8–10-week-old FVB/N mice). The mouse then recovered on a heating pad^75^. Following IC injection of cancer cells, mice were maintained under pathogen-free conditions. Two weeks postinjection, the mice were sacrificed. We then extracted their femurs and tibiae and maintained them in culture as described above. At this time point, the IC-injected mice began to look unhealthy and/or in pain. Per University policy and humane treatment of the animal subjects for research, the animals were sacrificed to avoid suffering. Upon examining the mice, we observed tumors in the thoracic cavity of some of the animals.

### Metastatic human bone samples

A femoral head was received from the Loyola Medical Center from a 59-year-old patient undergoing total hip arthroplasty due to breast cancer metastasis in the bone (IRB 15-04-2500). Cylindrical trabecular bone explants were excised using a diamond tip drill. Explants were fixed in 10% formalin for 24 h, decalcified in 10% EDTA, processed, and embedded in paraffin. Tissue was sliced into 5-μm sections and mounted on microscope slides for histology. For IHC staining of the human bone samples, rabbit IgG control was used at the same concentration as the CXCR2 antibody. In our analysis, “bone” refers to the portion of the section that is not marrow but could also include osteocytes, osteoblasts, osteoclasts, cancer cells, and/or any other cell that may have been recruited to this area during the progression of the disease.

### Cell viability assay

Cell viability in culture of the bone and marrow cells was quantified by CellTiter-Blue viability assay (Promega cat. # G8080) following the standard manufacturer’s protocol. Metabolic activity was quantified by measuring the fluorescence generated from metabolized resazurin (blue, non-fluorescent) that was converted into resorufin (pink, highly fluorescent) on a plate reader and compared to a fresh media control and to a fresh bone sample control. The fluorescence, which is proportional to the number of viable cells in the sample, was proportional to the number of bones contained in each well at the experimental endpoint. Fluorescence was measured using SpectraMax M3 (Molecular Devices) using 570 nm for excitation and 600 nm for measured emission using the system software (SoftMax Pro 6.2).

### Microscopy

Tissue sections stained by IHC were scanned by an Aperio CT scanner (Aperio Technologies) with a ×20 objective. Digital images were saved on the eSlide Manager database (version 12.3.2.5030). Images were manually inspected, analyzed, and annotated using the ImageScope software (Aperio Technologies).

Fluorescent images were taken with a Leica DM5500B microscope (Leica Microsystems) using a ×20 objective. We used the following fluorescent filters from Leica Microsystems: 4,6-diamidino-2-phenylindole for blue fluorescence (excitation 360 nm, excitation 470 nm), L5 for green fluorescence (excitation 480 nm, excitation 527 nm), and TXR for red fluorescence (excitation 560 nm, excitation 630 nm). Image acquisition was performed using the Leica Application Suite X (LAS X).

### In vivo imaging system (IVIS)

For visualization of the luciferase reporter gene that is expressed by the PyMT cancer cells, D-Luciferin (PerkinElmer cat. # 122796) was added directly in the media (for a total of 150 μg mL^−1^ working solution) of bone cultures immediately before imaging. Luciferase-positive regions were imaged using IVIS Lumina II (Caliper Life Sciences). The signal was analyzed using the system software (Living Image 4.2). The bones were subsequently fixed, demineralized, and embedded in paraffin for histology.

### Histology and tissue staining

Bone samples were fixed overnight in 4% PFA, stored in 70% ethanol prior to decalcification, decalcified for 2–3 weeks in 10% EDTA (EMD, cat. # EX0534-1) weight/volume at pH of 7.6, infused with paraffin in the tissue processor (Leica TP1020), paraffin embedded into blocks, and sectioned (4 μm) onto slides for histological analysis. The sections were all collected in the sagittal plane approximately half way through the block. One tissue section was examined per bone sample. The standard manufacturers’ protocols were followed for the following stains: H&E (Leica Biosystems, cat. # 3801571 and cat. # 3801606), TRAP (Sigma-Aldrich, cat. # 387A-1KT), trichrome (Sigma-Aldrich, cat. # HT15-1KT), and Safranin-O (Sigma-Aldrich, cat. # S2255). For detection of antigens that required antigen retrieval, sodium citrate antigen retrieval was conducted in a 95 °C water bath for 7 min prior to staining unless otherwise specified. The following antibodies were used for IHC: Pan-cytokeratin (0.26 μg mL^−1^ final; 1:750; Fisher Scientific, cat. # MS343P, uses Trypsin antigen retrieval), Ki67 (0.43 μg mL^−1^ final; 1:400; Cell Signaling, cat. # 9129 S), CD4 (2 μg mL^−1^ final; 1:250; Biorbyt, cat. # orb4830), CD8 (2 μg mL^−1^ final; 1:250; Biorbyt, cat. # orb182962), CD20 (0.8 μg mL^−1^ final; 1:250; Thermo Fisher Price, cat. # MA5-13141), CD68 (5 μg mL^−1^; 1:200; Bioss, cat. # bs-0649R), CXCL5 (10 μg mL^−1^ final; 1:50; R&D Systems, cat. # MAB433 in both mouse and human samples), CXCR2 (1.25 μg mL^−1^ final; 1:400; Abcam, cat. # ab14935 in both mouse and human samples), Ly6G/6 C (1.25 μg mL^−1^ final; 1:50; BD Pharmingen, cat. # 550291), Endomucin (4 μg mL^−1^ final; 1:50; Santa Cruz Biotechnology, cat. # sc-65495), CD61 (0.4 μg mL^−1^ final; 1:250; Cell Signaling, cat. # 13166), Cleaved-Caspase 3 (0.21 μg mL^−1^ final; 1:300; Cell Signaling, cat. # 9661), and CD71 (2 μg mL^−1^ final; 1:250; Invitrogen, cat. # 13-6800). One slide per bone sample was analyzed for each analysis, and the indicated number of animals and bone samples used are described in Table 1. Quality-control steps ensured that the samples were appropriate for analysis and included analyzing multiple fields of view across each sample, counting the total number of cells analyzed, and analyzing bone tissue samples collected at roughly the same depth per sample. In Table 1, we also included the number of cells analyzed per sample and per area analyzed.

### Quantification of Pan-keratin and Ki67 status in cancer cells

After staining bone co-culture samples by IHC for Pan-cytokeratin, the tissue sections were scanned using an Aperio ScanScope CS system to digitalize the images. Each image was analyzed by manually counting the number of double positive stained cells (Pan-cytokeratin and Ki67), and the percentage of double positive cells was calculated by dividing by the total number of Pan-cytokeratin-positive cells.

After double staining by IHC for Pan-cytokeratin and Ki67 (proliferation marker), the tissue sections were imaged and analyzed for the number of double positive stained cells (Pan-cytokeratin and Ki67), and the percentage of double positive cells was calculated by dividing by the total number of Pan-cytokeratin-positive cells. The number of cancer cells found per sample was somewhat variable. The majority of bone samples analyzed had between 700 and 1500 Keratin+ cells, while a few samples had <100 cells and a few others had >3000. The average number of Keratin+ cells across all samples used in the study was 1252.5 and the median was 1001 cells (Table 1). One section per bone sample was analyzed for the analysis. The sections were collected in the sagittal plane approximately half way through the bone. One researcher (R.R.-M.) analyzed and scored all of the samples blinded throughout the quantification. Samples damaged during the staining process or for which the staining was too weak to be scanned were excluded from this analysis.

### Cytokine and chemokine analysis

Cytokines and chemokines were analyzed by Mouse Discovery Assay (Eve Technologies, Mouse Cytokine Array/Chemokine Array 31-Plex). For each sample, conditioned media was collected as a supernatant of the media from bone and cancer cell cultures and processed by Eve Technologies according to the company’s specifications and requirements. Some samples measured zero, and some samples were considered out of range and not detectable. The out-of-range samples were given the value of zero for the analysis.

### Heatmap of cytokine and chemokine analysis

An Euclidian distance matrix was built using the data from the cytokine and chemokine Mouse Discovery Assay (Eve Technologies, Mouse Cytokine Array/Chemokine Array 31-Plex). Dendrograms were constructed using the Ward method on the distance matrix for the hierarchical clustering^76^. Samples for which measured concentration was zero across all compared samples of both cohorts were excluded from analysis. We used the publicly available R software^77^ for visualization of the heatmap.

### RNA extraction

Marrow from female FVB/N mice was collected by cutting both ends of femurs and tibiae followed by flushing the bone shafts with PBS using a 27-gauge needle. PyMT cancer cells were collected by trypsinization and serial washes with PBS. RNA was extracted using the RNA-Bee RNA isolation reagent (Amsbio, cat. # CS-104B) following the standard manufacturer’s protocol. Briefly, before adding RNA Bee, cultured cells were scraped from culture dishes and washed with PBS before flash freezing with liquid nitrogen. Cells and tissue were then homogenized with appropriate amounts of RNA Bee (1 mL per 50 mg tissue) on ice. Phase separation was carried out by adding 0.2 mL chloroform per 1 mL of RNA Bee, mixing, and centrifuging (12,000 × *g* for 15 min). RNA from the top aqueous layer is then precipitated with isopropanol, washed with 70% ethanol, dried, and dissolved in RNase-free water. RNA concentration and quality was measured with NanoDrop 2000 spectrometer (260/280 > 2.0).

### cDNA synthesis

cDNA synthesis from RNA was carried out with the QuantiTect Reverse Transcription Kit (Qiagen, cat. # 205311) following the standard manufacturer’s protocol. Briefly, 1 μg of RNA sample was measured out and genomic DNA removed by the included gDNA Wipeout Buffer. RT was then carried out in a 20 μL final volume with RT primer mix and reverse transcriptase. cDNA is then diluted to 400 μL, and 4 μL is used for each qPCR reaction.

### Quantitative polymerase chain reaction (qPCR)

qPCR from cDNA was carried out with 2× SYBR Green qPCR Master Mix (Bimake, cat. # B21203) following the standard manufacturer’s protocol. Primers for qPCR were ordered from Sigma-Aldrich (KiCqStart SYBR Green Primers). Primers’ sequences are as follows: CXCL5 (NM_009141): sense 5′-GGTCCACAGTGCCCTACG-3′ and anti-sense 5′-GCGAGTGCATTCCGCTTA-3′^78^, β-actin (NM_007393.5): sense 5′-GACCTCTATGCCAACACAGT-3′ and anti-sense 5′-AGTACTTGCGCTCAGGAGGA-3′. qPCR was carried out in a 20-μL reaction volume on 96-well Eppendorf twin.tec real-time PCR Plates (Eppendorf, cat. # 0030129636) with an Eppendorf Mastercycler eprealplex. No template controls and no primer controls are also carried out with each run. PCR products were ran in a 2% agarose gels to confirm amplification. CXCL5 expression was normalized to β-actin. We used bone marrow as the reference point to calculate fold increase.

### In situ hybridization

ISH of paraffin-embedded bone sections (prepared as described above for histology) used the following ISH probes: mouse CXCL5 (cat. # 467441), negative control probe (cat. # 310043). Antigen retrieval was performed using pretreatment kit from ACDbio on a hot plate at 100 °C for 15 min. Detection was performed using the RNAScope 2.5 HD Reagent Kit-RED (cat. # 322350) following the standard manufacturer’s protocols with the adapted antigen retrieval mentioned above and skipping the protease step. Following the ISH protocol, slides were incubated overnight at 4 °C in primary antibody anti-cytokeratin 8 (0.7 μg mL^−1^ final; 1:500; Abcam, cat. # ab53280) followed next day with a goat anti-rabbit Alexa-488-conjugated secondary antibody (8 μg mL^−1^ final; 1:250; Invitrogen, cat. # A11008) for 1 h at room temperature. Samples were counterstained with Hoechst 33342, cover-slipped, and imaged using the Leica DM5500B microscope as described above.

### Statistical analysis

Samples were compared statistically using the GraphPad Prism software (Version 6.0c) and R for Mac (R 3.1.1 GUI 1.65 Snow Leopard build). We compared each group pair by two-tailed unpaired Student’s *t* test when analyzing the number of double positive stained cells (Pan-cytokeratin and Ki67) and also when analyzing the average levels of CXCL5 in the media supernatant. For CellTiter-Blue assay analysis, we compared the samples by Student’s *t* test with the multiple comparisons function of the GraphPad Prism software.

### Reporting summary

Further information on research design is available in the Nature Research Reporting Summary linked to this article.

## Supplementary information

Supplementary figures

Reporting Summary

## Data Availability

The data that support the findings of this study are available within the article and its supplementary information files and from the corresponding author upon reasonable request.
