# Supplementary material for: The CXCL5/CXCR2 axis is sufficient to promote breast cancer colonization during bone metastasis
Source: Nat Commun. 2019 Sep 27;10:4404. doi: 10.1038/s41467-019-12108-6 (PMC6765048; doi:10.1038/s41467-019-12108-6)
Supplement: Supplementary file 1 — Supplementary figures [file 41467_2019_12108_MOESM1_ESM.pdf]

Supplementary information

Title

The CXCL5/CXCR2 axis is sufficient to promote breast cancer colonization during bone metastasis

Authors

Ricardo Romero-Moreno[1,2], Kimberly J. Curtis[1,3], Thomas R. Coughlin[1,3], Maria Cristina Miranda-Vergara[1,2], Shourik Dutta[1,2], Aishwarya Natarajan[1,2], Beth A. Facchine[1,2], Kristen M. Jackson[1,3], Lukas Nystrom[5], Jun Li[1,4], William Kaliney[1], Glen L. Niebur[1,3], and Laurie E. Littlepage[1,2,*]

Affiliations

[1]Harper Cancer Research Institute, South Bend, IN
[2]Department of Chemistry and Biochemistry, University of Notre Dame, Notre Dame, IN
[3]Tissue Mechanics Laboratory, Department of Aerospace and Mechanical Engineering, Bioengineering Graduate Program, University of Notre Dame, Notre Dame, IN
[4]Department of Applied and Computational Mathematics and Statistics, University of Notre Dame, Notre Dame, IN
[5]Department of Orthopaedic Surgery, Cleveland Clinic, Cleveland, OH

***Corresponding Author**

Laurie Littlepage, Ph.D.
Harper Cancer Research Institute, University of Notre Dame
1234 N Notre Dame Avenue, South Bend, IN 46617
Phone: (574) 631-4804 Fax: (574) 631-1165
Email: laurie.littlepage@nd.edu

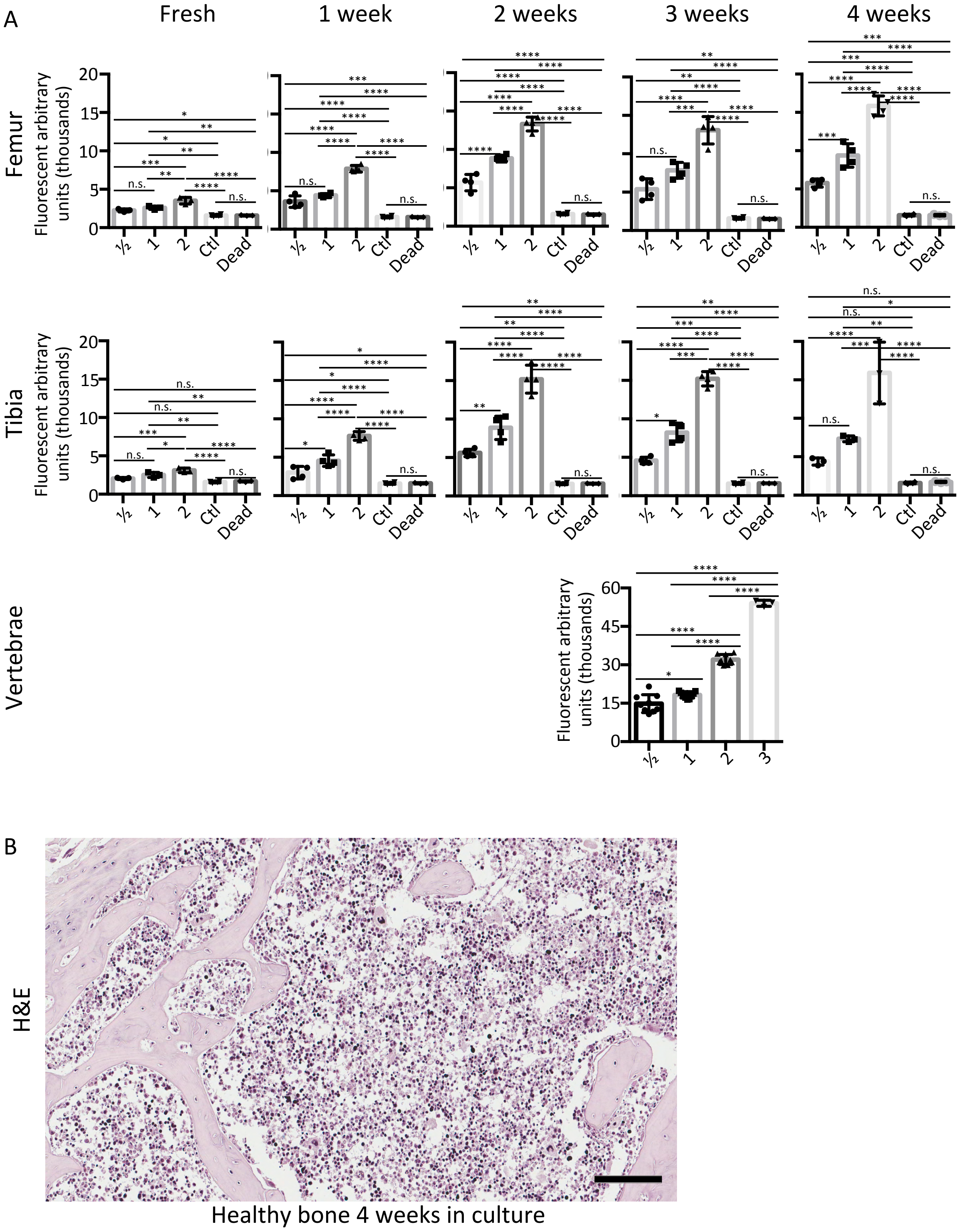

Supplementary figure 1

Supplementary Figure 1. (A) 4-week timeline of the metabolic activity of *ex vivo* bone cultures compared to the metabolic activity of a dead bone and media controls. CellTiter Blue (cell viability) assay to test metabolic activity of an increasing number of mouse bone explants in *ex vivo* culture up to four weeks in culture (p values obtained by student's t test, multiple comparisons). Lines show the mean and standard deviation. Please note that the different time points of the bone cultures were set up as staggered experiments so that the assay occurred on the same day for all of the timepoints using the same bottle of CellTiter Blue reagent and the same instrument settings. (B) H&E of a cultured mouse bone cultured for four weeks at a low magnification to show a wide area within the cultured bone (scale bar: 100 μm). * significance at $p<0.05$; ** significance at $p<0.01$; *** significance at $p<0.001$; **** significance at $p<0.0001$; n.s. not significant.

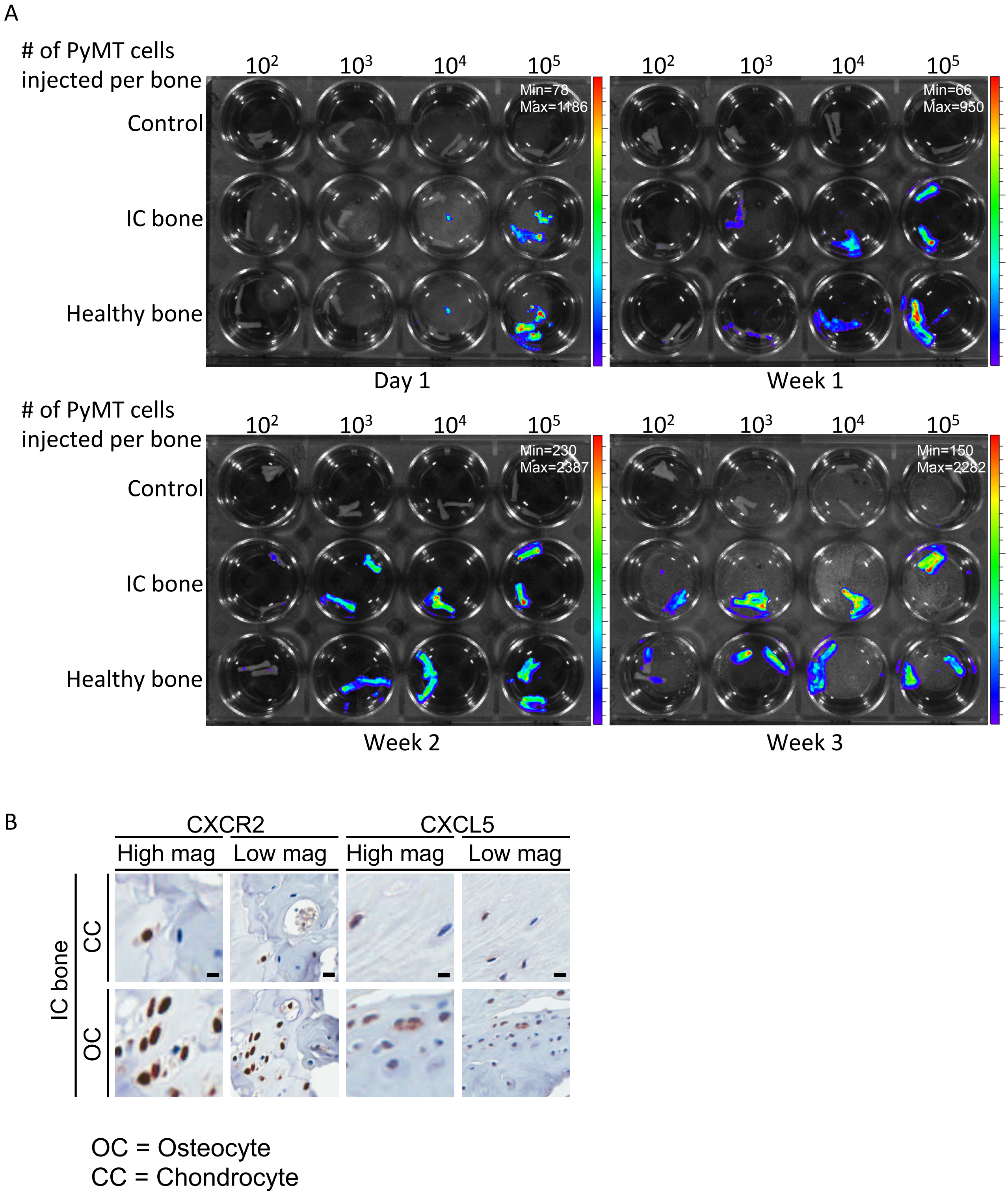

Supplementary figure 2

Supplementary Figure 2. (A) Luciferase assay on healthy or IC mouse bone cultures colonized by a luciferase-expressing PyMT cell line. The intensity bars (rainbow) and scale information (Min/max) for BLI signal are provided. Bioluminescent PyMT cancer cells co-localize with the bone in culture. (B) IHC staining for CXCR2 (left) and CXCL5 (right) of IC bone co-cultures injected with PyMT cells. We are showing that chondrocytes (CC) and osteocytes (OC) are capable of expressing both CXCR2 and CXCL5 and these are not restricted to cells in the bone marrow or to cancer cells (scale bars: High mag = 5 μm; Low mag = 10 μm).
