# Supplementary material for: The CXCL5/CXCR2 axis is sufficient to promote breast cancer colonization during bone metastasis
Source: Nat Commun. 2019 Sep 27;10:4404. doi: 10.1038/s41467-019-12108-6 (PMC6765048; doi:10.1038/s41467-019-12108-6)
Supplement: Supplementary file 2 — Reporting Summary [file 41467_2019_12108_MOESM2_ESM.pdf]

nature research

Corresponding author(s): Laurie Littlepage, Ph.D.

Last updated by author(s): August 7, 2019

Reporting Summary

Nature Research wishes to improve the reproducibility of the work that we publish. This form provides structure for consistency and transparency in reporting. For further information on Nature Research policies, see Authors & Referees and the Editorial Policy Checklist.

Statistics

For all statistical analyses, confirm that the following items are present in the figure legend, table legend, main text, or Methods section.

n/a	Confirmed	
☐	☒	The exact sample size (n) for each experimental group/condition, given as a discrete number and unit of measurement
☐	☒	A statement on whether measurements were taken from distinct samples or whether the same sample was measured repeatedly
☐	☒	The statistical test(s) used AND whether they are one- or two-sided<br>*Only common tests should be described solely by name; describe more complex techniques in the Methods section.*
☒	☐	A description of all covariates tested
☐	☒	A description of any assumptions or corrections, such as tests of normality and adjustment for multiple comparisons
☐	☒	A full description of the statistical parameters including central tendency (e.g. means) or other basic estimates (e.g. regression coefficient) AND variation (e.g. standard deviation) or associated estimates of uncertainty (e.g. confidence intervals)
☐	☒	For null hypothesis testing, the test statistic (e.g. F, t, r) with confidence intervals, effect sizes, degrees of freedom and P value noted<br>*Give P values as exact values whenever suitable.*
☒	☐	For Bayesian analysis, information on the choice of priors and Markov chain Monte Carlo settings
☒	☐	For hierarchical and complex designs, identification of the appropriate level for tests and full reporting of outcomes
☒	☐	Estimates of effect sizes (e.g. Cohen's d, Pearson's r), indicating how they were calculated

Our web collection on statistics for biologists contains articles on many of the points above.

Software and code

Policy information about availability of computer code

Data collection	ImageScope Software (Aperio Technologies)
Data analysis	For data analysis: R statistical language was used (version 2014).

For manuscripts utilizing custom algorithms or software that are central to the research but not yet described in published literature, software must be made available to editors/reviewers. We strongly encourage code deposition in a community repository (e.g. GitHub). See the Nature Research guidelines for submitting code & software for further information.

Data

Policy information about availability of data

All manuscripts must include a data availability statement. This statement should provide the following information, where applicable:

- Accession codes, unique identifiers, or web links for publicly available datasets
- A list of figures that have associated raw data
- A description of any restrictions on data availability

The data that support the findings of this study are available within the article and its supplementary information files and from the corresponding author upon reasonable request.

Field-specific reporting

Please select the one below that is the best fit for your research. If you are not sure, read the appropriate sections before making your selection.

☒ Life sciences ☐ Behavioural & social sciences ☐ Ecological, evolutionary & environmental sciences

Life sciences study design

All studies must disclose on these points even when the disclosure is negative.

Sample size	The sample sizes for each experiment are included in Table 1. For our experiments, we aimed to have four or more bone cultures represented in the data for each experiment. We also used multiple mice and multiple bones per mouse (e.g., femur and tibia, each cut in half). While our study did not use a large number of animals, each condition had a significant number of separate bone culture samples (typically 5-11 samples per cohort) analyzed individually. These samples were separately cultured, even when from the same animal, and should be considered biological replicates, not technical replicates, because each bone sample was manually and individually injected with cancer cells, grown in separate wells for each bone, and analyzed separately and blindly. Each cultured sample had the potential for experimental variability. For histological analysis, multiple fields of view were analyzed across the samples, and quality control steps were taken to make sure that the samples were appropriate for analysis. Every effort was made to analyze bone tissue samples collected at roughly the same depth per sample.
Data exclusions	Samples damaged during the staining process or for which the staining was too weak to be scanned were excluded from this analysis.
Replication	IHC staining was often done on consecutive slides of the same sample giving similar results. Different culture conditions were used on technical and biological replicates giving similar results.
Randomization	Mice for experimentation for each experiment were chosen based on their date of birth and no particular measurement was taken to allocate them in experimental groups, purely at random.
Blinding	Researchers were blinded when conducting manual analysis of the samples.

Reporting for specific materials, systems and methods

We require information from authors about some types of materials, experimental systems and methods used in many studies. Here, indicate whether each material, system or method listed is relevant to your study. If you are not sure if a list item applies to your research, read the appropriate section before selecting a response.

Materials & experimental systems

n/a	Involved in the study	
☐	☒	Antibodies
☐	☒	Eukaryotic cell lines
☒	☐	Palaeontology
☐	☒	Animals and other organisms
☒	☐	Human research participants
☒	☐	Clinical data

Methods

n/a	Involved in the study	
☒	☐	ChIP-seq
☒	☐	Flow cytometry
☒	☐	MRI-based neuroimaging

Antibodies

Antibodies used	The following antibodies were used for immunohistochemistry (IHC): Pan-cytokeratin (Fisher Scientific, cat. # MS343P, uses Trypsin antigen retrieval), Ki67 (Cell Signaling, cat. # 9129S), CD4 (Biorbyt, cat. # orb4830), CD8 (Biorbyt, cat. # orb182962), CD20 (Thermo Fisher Price, cat. # MA5-13141), CD68 (Bioss, cat. # bs-0649R), CXCL5 (R&D Systems, cat # MAB433 in both mouse and human samples), CXCR2 (Abcam, cat # ab14935 in both mouse and human samples), Ly6G/6C (BD Pharmingen, cat # 550291), Endomucin (Santa Cruz Biotechnology, cat # sc-65495), CD61 (Cell Signaling, cat # 13166), Cleaved-Caspase 3 (Cell Signaling, cat # 9661), and CD71 (Invitrogen, cat # 13-6800).
Validation	The antibody validation for each antibody is described at the manufacturers' websites and, in some cases when appropriate, confirmed by pathology.

Eukaryotic cell lines

Policy information about cell lines

Cell line source(s)	Obtained from Conor Lynch. Halpern, J. et al. The application of a murine bone bioreactor as a model of tumor: bone interaction. Clin. Exp. Metastasis 23, 345–356 (2007).
Authentication	Our cell line was authenticated prior to the initial experiments using STR profiles (Genetica cell line testing).
Mycoplasma contamination	The cell line tested negative for mycoplasma contamination using a mycoplasma detection kit (InvivoGen, cat # rep-pt1).
Commonly misidentified lines (See ICLAC register)	We did not use a commonly misidentified cell line in this study.

Animals and other organisms

Policy information about studies involving animals; ARRIVE guidelines recommended for reporting animal research

Laboratory animals	We used FVB/N mice for this study (Mus musculus). Mice were acquired from Harlan Laboratories, Inc. (now Envigo). All female 8-15 weeks of age at the moment we sacrificed them.
Wild animals	This study did not use wild animals.
Field-collected samples	This study did not involve samples collected in the field
Ethics oversight	Animal experiments were conducted with approval from the University of Notre Dame Institutional Animal Care and Use Committee (IACUC) for the ethical treatment of animals (protocol # 15-10-2724). The human research was approved by IRB (15-04-2500).

Note that full information on the approval of the study protocol must also be provided in the manuscript.
